# Two venom allergen‐like proteins, HaVAP1 and HaVAP2, are involved in the parasitism of *Heterodera avenae*

**DOI:** 10.1111/mpp.12768

**Published:** 2019-01-09

**Authors:** Shujie Luo, Shiming Liu, Lingan Kong, Huan Peng, Wenkun Huang, Heng Jian, Deliang Peng

**Affiliations:** ^1^ State Key Laboratory for Biology of Plant Diseases and Insect Pests, Institute of Plant Protection Chinese Academy of Agricultural Sciences Beijing 100193 China; ^2^ Key Laboratory of Plant Pathology of Ministry of Agriculture, College of Plant Protection China Agricultural University Beijing 100193 China

**Keywords:** CYPRO4‐like protein, HaVAP1, HaVAP2, *Heterodera avenae*, *Hordeum vulgare*, venom allergen‐like protein

## Abstract

Despite the fact that venom allergen‐like proteins (VAPs) have been identified in many animal‐ and plant‐parasitic nematodes, studies on VAPs in *Heterodera avenae*, which is an important phytonematode, are still in their infancy. Here, we isolated, cloned and characterized two *VAP*s, named *HaVAP1* and *HaVAP2*, from *H. avenae*. The two encoded proteins, HaVAP1 and HaVAP2, harbour an SCP‐like domain each, but share only 38% identity with each other. *HaVAP1* and *HaVAP2* are expressed in subventral and dorsal oesophageal glands, respectively. *HaVAP1* is expressed mainly at the early stages, whereas *HaVAP2* accumulates principally at the late stages. Both HaVAP1 and HaVAP2 are secreted when expressed in *Nicotiana benthamiana *leaves, but HaVAP1 is delivered into chloroplasts, whereas HaVAP2 is translocated to the nucleus without signal peptides. Knocking down *HaVAP1* increased the virulence of *H. avenae*. In contrast, silencing of *HaVAP2* hampered the parasitism of *H. avenae*. Both HaVAP1 and HaVAP2 suppressed the cell death induced by BAX in *N. benthamiana* leaves. Moreover, HaVAP2 physically interacted with a CYPRO4‐like protein (HvCLP) of *Hordeum vulgare* in the nucleus of the plant. It is reasonable to speculate that the changes in the transcript of *HvCLP* are associated with *HaVAP2* during the parasitism of *H. avenae*. All results obtained in this study show that both HaVAP1 and HaVAP2 are involved in the parasitism of *H. avenae*, but they possess different functions, broadening our understanding of the parasitic mechanism of *H. avenae*.

## Introduction

Phytonematodes, as well as other pathogens, are serious threats to plants worldwide. The main differences between phytonematodes and other pathogens are that phytonematodes are animals and possess specific structures, such as stylets, amphids and secretory gland cells, for parasitism in plants (Baldwin *et al.*, 2004; Davis *et al.*, 2004). Different from migratory endoparasitic nematodes, sedentary endoparasitic nematodes induce and establish permanent feeding sites as sole nutrition sources (Hussey and Grundler, 1998). Effectors secreted from oesophageal gland cells, amphids and cuticles play important roles in the parasitism of phytonematodes (Haegeman *et al.*, 2012). There are three general categories of effectors: first, cell wall‐degrading enzymes (CWDEs), which loosen or degrade the cell wall for nematodes to pierce and colonize plants; second, metabolism‐associated effectors, which induce the formation and maintenance of feeding sites for compatible interactions; and third, effectors that are used for the suppression of plant resistance responses (Ali *et al.*, 2017). Based on the plant immune system (Jones and Dangl, 2006), the effectors function in two ways, termed effector‐triggered susceptibility (ETS) and effector‐triggered immunity (ETI) (Hewezi and Baum, 2013). If an effector recognized by the host resistance system triggers ETI during parasitism, this effector is called an avirulence (Avr) protein (Desveaux *et al.*, 2006). In contrast, most effectors are recognized as virulence proteins which promote compatible interactions with their hosts (Davis *et al.*, 2008; Rosso *et al.*, 2011).

*Heterodera avenae* (cereal cyst nematode, CCN) is a type of sedentary endoparasitic nematode. Its hosts include wheat, barley, oats and several other cereal crops. Recently, an increasing number of effectors have been identified from *H. avenae.* For instance, Long *et al. *(2013) isolated two β‐1,4‐endoglucanase genes and analysed their functions in the parasitism of *H. avenae*. In 2014, an acid phosphatase gene (*Ha‐acp1*) from *H. avenae* was characterized (Liu *et al.*, 2014). Chen C *et al. *(2015) identified an annexin‐like protein from *H. avenae* and confirmed its positive effect during parasitism. A fatty acid‐ and retinol‐binding protein, which is able to bind fatty acids and retinol, was isolated from *H. avenae* (Le *et al.*, 2016). Liu *et al. *(2016) identified an expansin‐like protein from *H. avenae* and verified its role in the promotion of parasitism. Furthermore, transcriptome sequencing and *in silico* analyses revealed many novel putative effectors from *H. avenae* (Chen C *et al.*, 2017, 2018; Kumar *et al.*, 2014; Yang *et al.*, 2017; Zheng *et al.*, 2015). However, except for an initial study by Chen *et al. *(2018), little information is available on the venom allergen‐like proteins (VAPs) of *H. avenae*.

VAPs are a type of cysteine‐rich secretory protein belonging to the sperm coating protein/Tpx‐1/Ag‐5/Pr‐1/Sc‐7 (SCP/TAPS) superfamily (Cantacessi and Gasser, 2012; Cantacessi *et al.*, 2009). VAPs have been isolated from all plant‐ and animal‐parasitic nematodes (PPNs and APNs) (Jasmer *et al.*, 2003). As early as 1999, a venom allergen antigen 5‐like protein‐coding gene, named *Mi‐msp‐1*, was cloned from *Meloidogyne incognita* (Ding *et al.*, 2000). Southern blotting analysis indicated that *Meloidogyne arenaria* and *Meloidogyne javanica* might harbour *Mi‐msp‐1 *homologues. Gao *et al. *(2001) analysed and characterized two *VAP* genes in *Heterodera glycines*. Although the VAPs are universal in nematodes, there were few studies on the functions of these genes in infections by phytonematodes until 2012. GrVAP1, which was isolated from *Globodera rostochiensis*, interacts with the apoplastic papain‐like cysteine protease Rcr3^pim^. Perturbation of Rcr3^pim^ activates the Cf‐2‐mediated resistance of *Solanum pimpinellifolium* to *G. rostochiensis* (Lozano‐Torres *et al.*, 2012). Nevertheless, Rcr3^pim^ promotes infection by *G. rostochiensis* when expressed in *S. pimpinellifolium* without Cf‐2 (Lozano‐Torres *et al.*, 2012). Further study revealed that the VAPs isolated from *G. rostochiensis *and *Heterodera schachtii* suppressed basal host innate immunity to facilitate the pathogenicity of nematodes and other unrelated pathogens by perturbing surface‐localized immune receptors (Lozano‐Torres *et al.*, 2014).

In spite of the wide range and conservation of VAPs in nematodes, little is known about the details of this type of protein in *H. avenae*. In this study, two *VAP* homologues were isolated and cloned from *H. avenae* based on an expressed sequence tag (EST) library. Because of the significantly low sequence identity shared between these two VAP homologues, we treated them as two independent genes during this research. Temporal and spatial expression patterns verified the gap between these genes. Both transient expression and RNA interference (RNAi) results showed their different functions during infection. To uncover the virulence targets of these two proteins, yeast two‐hybrid (Y2H) screening was carried out using *Hordeum vulgare* root cDNA as prey. Our results indicate the different roles of these two VAPs in the parasitism of *H. avenae*.

## Results

### Cloning and sequence analyses of two *VAP* genes

Two *VAP* genes were cloned from *H. avenae* by rapid amplification of cDNA ends (RACE). They were designated *HaVAP1* (GenBank accession MH255798) and *HaVAP2* (GenBank accession MH255799). *HaVAP1* contains a full‐length cDNA of 919 bp that includes a coding sequence (CDS) of 657 bp (Fig. S1a, see Supporting Information). The full‐length cDNA and CDS of *HaVAP2* are 879 and 651 bp long, respectively (Fig. S2a, see Supporting Information). The *HaVAP1* cDNA encodes a 218‐amino‐acid protein with an N‐terminal signal peptide of 25 amino acids for secretion, according to the SignalP 4.1 server (Fig. S1b). The encoded protein and the corresponding N‐terminal signal peptide of HaVAP2 contain 216 amino acids and 23 amino acids, respectively (Fig. S2b). The CD‐search from the National Center for Biotechnology Information (NCBI) identified a putative SCP‐like extracellular protein domain which is located from peptide position 34 to 180 in HaVAP1 (Fig. S1b). Another SCP‐like extracellular protein domain was also found to be located from peptide position 38 to 176 in HaVAP2 using the same method (Fig. S2b). Based on the pI/Mw prediction from the ExPASy program, HaVAP1 has a molecular mass of 23.8 kDa and a theoretical isoelectric point of 7.98, whereas HaVAP2 has a molecular mass of 23.8 kDa and a theoretical isoelectric point of 5.77. The *HaVAP1* gDNA shows four introns, having lengths of 90, 208, 55 and 124 bp, respectively, which separate the CDS into five exons with lengths of 52, 279, 119, 95 and 112 bp, respectively (Fig. S1a). The *HaVAP2* gDNA consists of four introns (60, 190, 45 and 467 bp) and five exons (43, 185, 94, 211 and 118 bp) (Fig. S2a).

**Figure 1:**
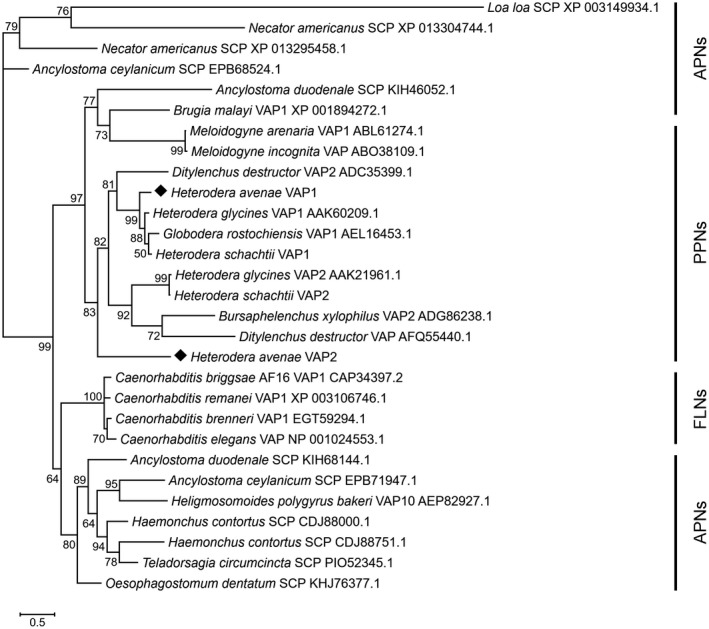
Maximum likelihood phylogenetic tree of HaVAP1, HaVAP2 (marked with rhombi) and 27 homologues from plant‐parasitic nematodes (PPNs), animal‐parasitic nematodes (APNs) and free‐living nematodes (FLNs) at the amino acid level. The Shimodaira–Hasegawa local support given at each node is inferred from 1000 replicates. The accession number of each protein used in this tree is attached to the name of the protein, except for *Heterodera schachtii* VAP1 and VAP2, which are derived from Lozano‐Torres *et al*. (2014).

A BLASTp search revealed many homologues of VAPs or SCP‐like proteins at the amino acid level in other nematodes. HaVAP1 shares identity with VAPs from *Globodera rostochiensis* (75%), *Heterodera glycines* (71%) and *Ditylenchus destructor* (48%). In contrast, the sequence alignment analysis of HaVAP2 showed a low degree of similarity with VAPs or SCP‐like proteins from other organisms. Only the SCP‐like protein of *Oesophagostomum dentatum*, an APN, shares more than 40% identity with HaVAP2. The VAPs from PPNs, such as *G. rostochiensis*, *D. destructor* and *H. glycines*, share identities of only 39%, 38% and 36%, respectively, with HaVAP2. The amino acid sequence identity is 38% between HaVAP1 and HaVAP2. A maximum likelihood phylogenetic tree (Fig. 1) was constructed to analyse the relationships among HaVAP1, HaVAP2 and 27 homologues from PPNs, APNs and free‐living nematodes (FLNs) at the amino acid level. Among all the homologues, 18 proteins have the annotation of VAP and the other nine proteins harbour SCP‐like domains. The phylogenetic tree (Fig. 1) shows that HaVAP1 is first clustered with *H. schachtii* VAP1, *G. rostochiensis* VAP1, *H. glycines* VAP1 and *D. destructor* VAP2 as a monophyletic clade, then successively sisters with the group comprising *D. destructor* VAP, *Bursaphelenchus xylophilus* VAP2, *H. glycines* VAP2 and *H. schachtii* VAP2. Unlike HaVAP1, HaVAP2 is far from the two clades mentioned above.

### *HaVAP1* and *HaVAP2* show different tissue localizations and different expression patterns during parasitism

**Figure 2:**
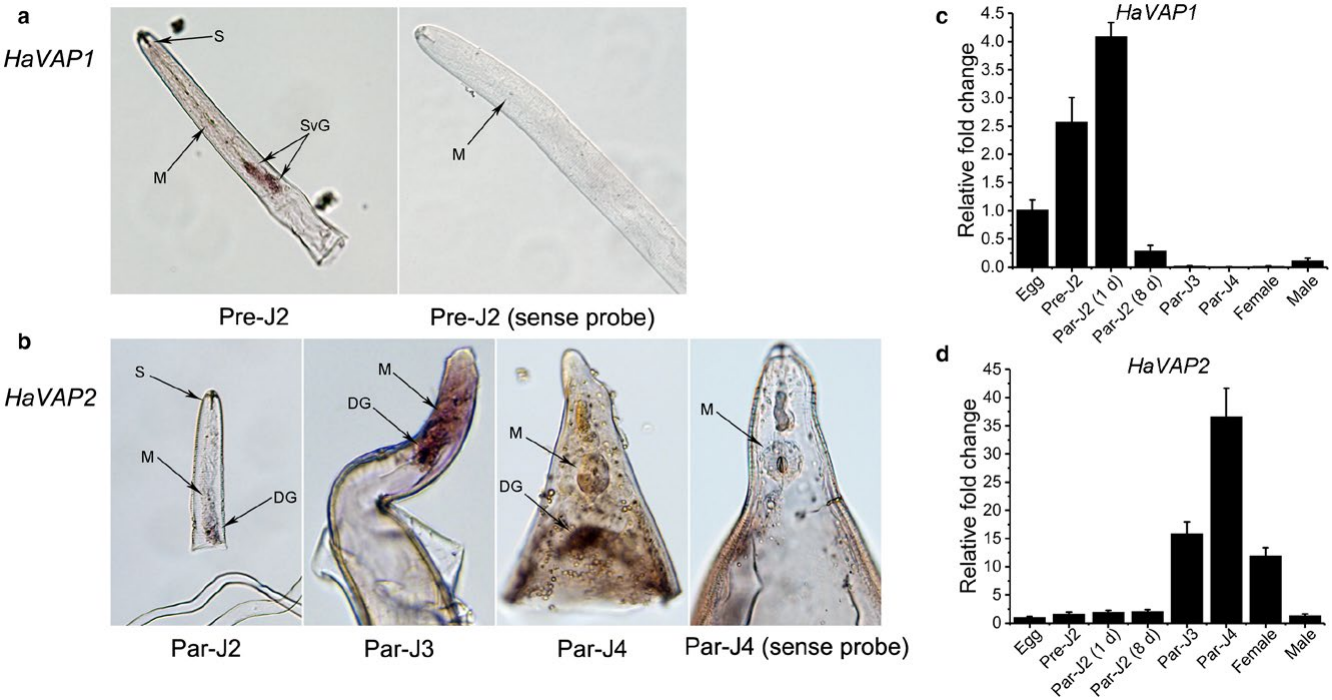
Tissue localization and developmental expression pattern analyses of *HaVAP1* and *HaVAP2* using *in situ* hybridization and quantitative reverse transcription‐polymerase chain reaction (qRT‐PCR), respectively. (a) *HaVAP1* located in the subventral oesophageal glands (SvG) of pre‐parasitic second‐stage juvenile (pre‐J2) after hybridization with antisense probe. (b) *HaVAP2* located in the dorsal oesophageal glands (DG) of parasitic J2 (par‐J2), parasitic third‐stage juvenile (par‐J3) and parasitic fourth‐stage juvenile (par‐J4). No signals were observed after hybridization with sense probes. M, metacorpal bulb; S, stylet. Transcript level analyses of *HaVAP1* (c) and *HaVAP2* (d) at seven different developmental stages, including egg, pre‐J2, par‐J2 at 1 day after inoculation [par‐J2 (1 d)], par‐J2 at 8 days after inoculation [par‐J2 (8 d)], par‐J3, par‐J4 and adult (female and male). The fold change values and bars represent the means and standard deviations (SD), respectively.

*In situ* hybridization assays were used to detect the tissue localizations of transcripts of *HaVAP1* and *HaVAP2* in juveniles. Significant signals were observed in the subventral oesophageal gland cells of pre‐parasitic second‐stage juveniles (pre‐J2s) using a digoxigenin (DIG)‐labelled antisense probe designed for *HaVAP1* (Fig. 2a). The transcript of *HaVAP2* mainly accumulated in the dorsal oesophageal glands of parasitic J2s (par‐J2s), parasitic third‐stage juveniles (par‐J3s) and parasitic fourth‐stage juveniles (par‐J4s) (Fig. 2b). No signals were observed in juveniles hybridized with sense probes (Fig. 2a,b).

We used quantitative reverse transcription‐polymerase chain reaction (qRT‐PCR) to quantify the transcript levels of *HaVAP1* and *HaVAP2* at seven *H. avenae* developmental stages, including egg, pre‐J2, par‐J2 1 day after inoculation [par‐J2 (1 d)], par‐J2 8 days after inoculation [par‐J2 (8 d)], par‐J3, par‐J4 and adult (female and male). The transcript levels at the egg stage were taken as the standard values. The *HaVAP1* transcript level increased significantly from egg to pre‐J2 and par‐J2 (1 d). Then, a marked decrease occurred at par‐J2 (8 d). At the stages of par‐J3, par‐J4 and female, *HaVAP1* transcript abundances were reduced to their lowest point. However, a weak rise in the transcript abundance of *HaVAP1* was observed at the male stage (Fig. 2c). *HaVAP2* transcripts accumulated in par‐J3, par‐J4 and female. In particular, *HaVAP2* peaked at approximately 36 times more transcripts at the par‐J4 stage than at the egg stage. The other stages, including egg, pre‐J2, par‐J2 (1 d), par‐J2 (8 d) and male, all showed low levels of transcript of *HaVAP2* (Fig. 2d).

### HaVAP1 and HaVAP2 are both secreted proteins, but show different subcellular localizations without signal peptides

**Figure 3:**
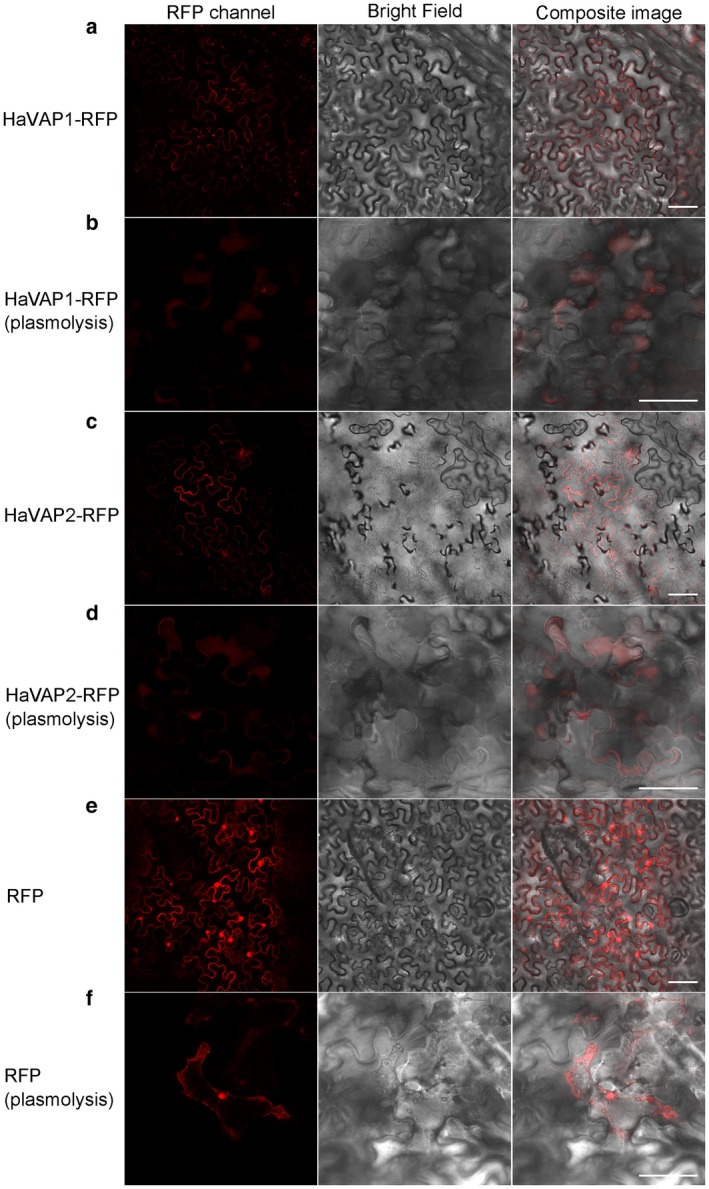
Subcellular localization of HaVAP1‐RFP and HaVAP2‐RFP in *Nicotiana benthamiana* leaves using agroinfiltration. HaVAP1 (a) and HaVAP2 (c) were fused to the N‐terminus of a red fluorescent protein (RFP) reporter and accumulated in the cell membrane or cell wall. Plasmolysis assays based on the expression of HaVAP1‐RFP (b) and HaVAP2‐RFP (d) showed that signals were observed between the cell membrane and cell wall. (e) Free RFP derived from empty vector accumulated in whole cells. (f) Free RFP accumulated in protoplast after plasmolysis. All signals were collected at 3 days after agroinfiltration. Bars, 50 μm.

A red fluorescent protein (RFP) reporter was fused to the C‐terminus of either HaVAP1 or HaVAP2 and transiently expressed in *Nicotiana benthamiana*. Subcellular localization assays showed that both HaVAP1‐RFP (Fig. 3a) and HaVAP2‐RFP (Fig. 3c) localized on the cell membrane or cell wall at 3 days after infiltration. To discriminate the exclusive localizations, we conducted plasmolysis assays with 30% glycerine–water solution by infiltration. The extracellular signals of HaVAP1‐RFP (Fig. 3b) and HaVAP2‐RFP (Fig. 3d) were observed between the cell membrane and cell wall after plasmolysis. As a control, free RFP accumulated in the protoplast before (Fig. 3e) or after (Fig. 3f) plasmolysis. Such a phenomenon, in which HaVAP1 and HaVAP2 localized in the extracellular space, confirms that the signal peptides in both proteins are indeed able to target the proteins for secretion.

**Figure 4:**
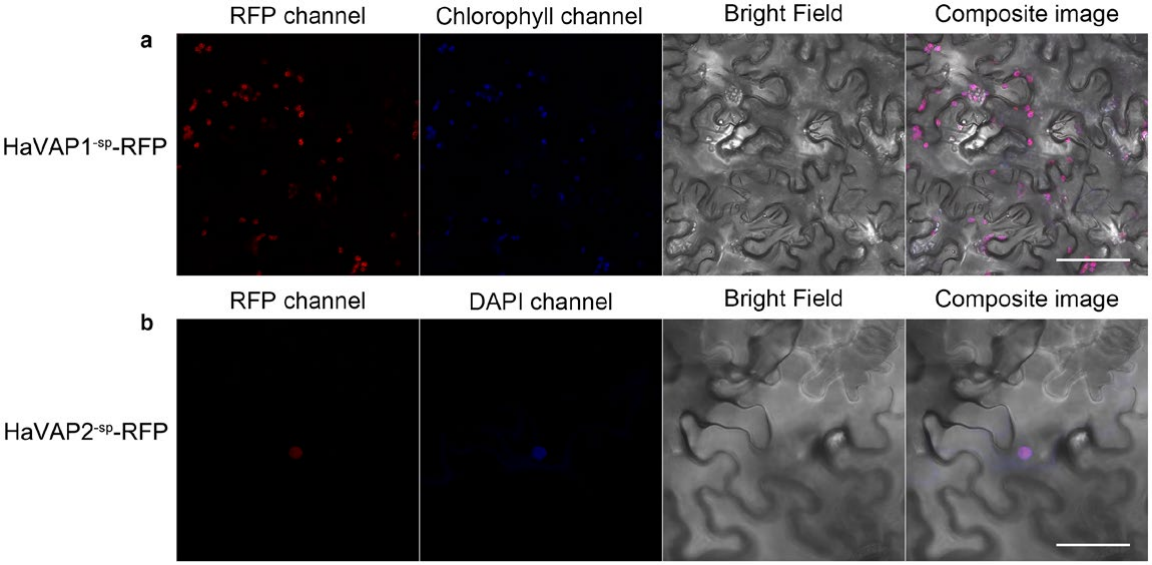
Subcellular localization of HaVAP1^‐sp^‐RFP and HaVAP2^‐sp^‐RFP in *Nicotiana benthamiana* leaves after agroinfiltration. (a) HaVAP1^‐sp^ (minus signal peptide amino acid sequence) was fused to the N‐terminus of a red fluorescent protein (RFP) reporter and accumulated in chloroplasts (autofluorescence). (b) HaVAP2^‐sp^ was fused to the N‐terminus of an RFP reporter and accumulated in the nucleus, which was counterstained with the nucleic acid stain 4′,6‐diamidino‐2‐phenylindole (DAPI). All the signals were collected at 3 days after agroinfiltration. Bars, 50 μm.

Subsequently, we conducted the fusion of HaVAP1^‐sp^‐RFP and HaVAP2^‐sp^‐RFP to analyse the subcellular localization of both proteins without signal peptides in *N. benthamiana*. As shown in Fig. 4a, HaVAP1^‐sp^‐RFP accumulated in chloroplasts, and the corresponding signals merged with the chlorophyll signals at 3 days after infiltration. However, a different signal was captured when *Agrobacterium tumefaciens* carrying HaVAP2^‐sp^‐RFP was infiltrated into *N. benthamiana* leaves. After confirmation, HaVAP2^‐sp^‐RFP accumulated in the nucleus, and the corresponding signal merged with the signal from the nucleic acid stain 4′,6‐diamidino‐2‐phenylindole (DAPI) (Fig. 4b).

### *In vitro* RNAi of *HaVAP1* promotes the onset of parasitism by *H. avenae*

**Figure 5:**
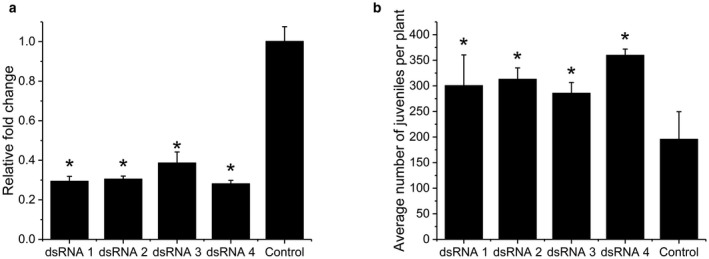
*In vitro* RNA interference (RNAi) of *HaVAP1* promotes the onset of parasitism by *Heterodera avenae*. (a) Quantitative reverse transcription‐polymerase chain reaction (qRT‐PCR) showed that all four double‐stranded RNAs (dsRNAs) knocked down *HaVAP1* transcript levels in pre‐parasitic second‐stage juveniles of *H. avenae* after 36 h of soaking. The fold change values and bars represent the means and standard deviation (SD), respectively. Asterisks (*) indicate significant differences according to Student’s *t*‐test, *P* < 0.01. (b) Treatments with all four dsRNAs significantly increased the numbers of juveniles of *H. avenae* in plant roots compared with the control at 8 days after inoculation. The average numbers and bars represent the means and SD, respectively. Asterisks (*) indicate significant differences according to Student’s *t*‐test, *P* < 0.05. Juveniles soaked in dsRNA solution in which dsRNA was replaced with diethyl pyrocarbonate (DEPC)‐treated water were used as control.

To investigate whether *HaVAP1* is required for the parasitism of *H. avenae*, an *in vitro* RNAi experiment was conducted by soaking juveniles in double‐stranded RNAs (dsRNAs) targeting *HaVAP1*. The qRT‐PCR assay showed significant reductions in *HaVAP1* transcripts after RNAi (Fig. 5a). Soaking the juveniles in dsRNAs did not result in significant changes in their survival rates compared with the juveniles in the control (data not shown). Next, the treated pre‐J2s were inoculated onto susceptible barley plants (*H. vulgare* cultivar Golden Promise). Eight days after inoculation, all four treatments with dsRNAs significantly increased the numbers of juveniles in *H. vulgare* roots by about 46%–84% compared with the control plants (Fig. 5b). These results indicate that *in vitro* RNAi of *HaVAP1* promotes the onset of parasitism by *H. avenae*.

### *HaVAP2* is required for the parasitism of *H. avenae*

**Figure 6:**
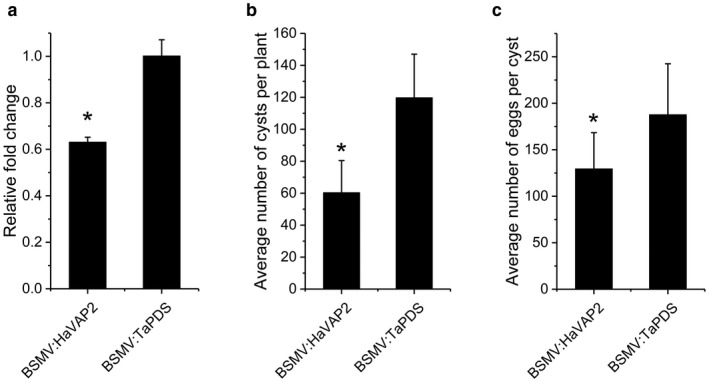
HaVAP2 promotes the parasitism of *Heterodera avenae*. (a) Quantitative reverse transcription‐polymerase chain reaction (qRT‐PCR) showed that the transcript abundance of *HaVAP2* was inhibited in *H. avenae* collected from BSMV:*HaVAP2*‐treated plants relative to *H. avenae* collected from BSMV:*TaPDS*‐treated plants at 40 days after inoculation. The fold change values and bars represent the means and standard deviation (SD), respectively. (b) Fewer cysts per plant were observed in BSMV:*HaVAP2*‐treated plants than in BSMV:*TaPDS*‐treated plants at 90 days after inoculation. The average numbers and bars represent the means and SD, respectively. (c) Fewer eggs per cyst were collected from BSMV:*HaVAP2*‐treated plants than from BSMV:*TaPDS*‐treated plants. The average numbers and bars represent the means and SD, respectively, of 20 independent repeats. Asterisks (*) indicate significant differences according to Student’s *t*‐test, *P* < 0.01.

Because *HaVAP2* transcripts accumulate in par‐J3, par‐J4 and female, we selected *Barley stripe mosaic virus* (BSMV) virus‐induced gene silencing (VIGS) to investigate whether *HaVAP2* is required for the parasitism of *H. avenae*. Forty days after the inoculation of viruses and nematodes, qRT‐PCR analysis was performed, and showed that the transcript abundance of *HaVAP2* was significantly lower in females from BSMV:*HaVAP2*‐treated wheat (*Triticum aestivum* cultivar WEN19) than from BSMV:*TaPDS*‐treated wheat (Fig. 6a) plants. The average number of cysts isolated from BSMV:*HaVAP2*‐treated plants was approximately 50% lower than that from BSMV:*TaPDS*‐treated plants at 90 days after inoculation (Fig. 6b). To further verify the role of HaVAP2 in nematode reproduction, we counted the numbers of eggs in the cysts collected above. The egg number in BSMV:*HaVAP2*‐treated plants was *c*. 31% less than that in BSMV:*TaPDS*‐treated plants (Fig. 6c). These findings suggest that *HaVAP2* is required for the parasitism of *H. avenae*.

### Both HaVAP1 and HaVAP2 suppress BAX‐induced cell death in *N. benthamiana*

**Figure 7:**
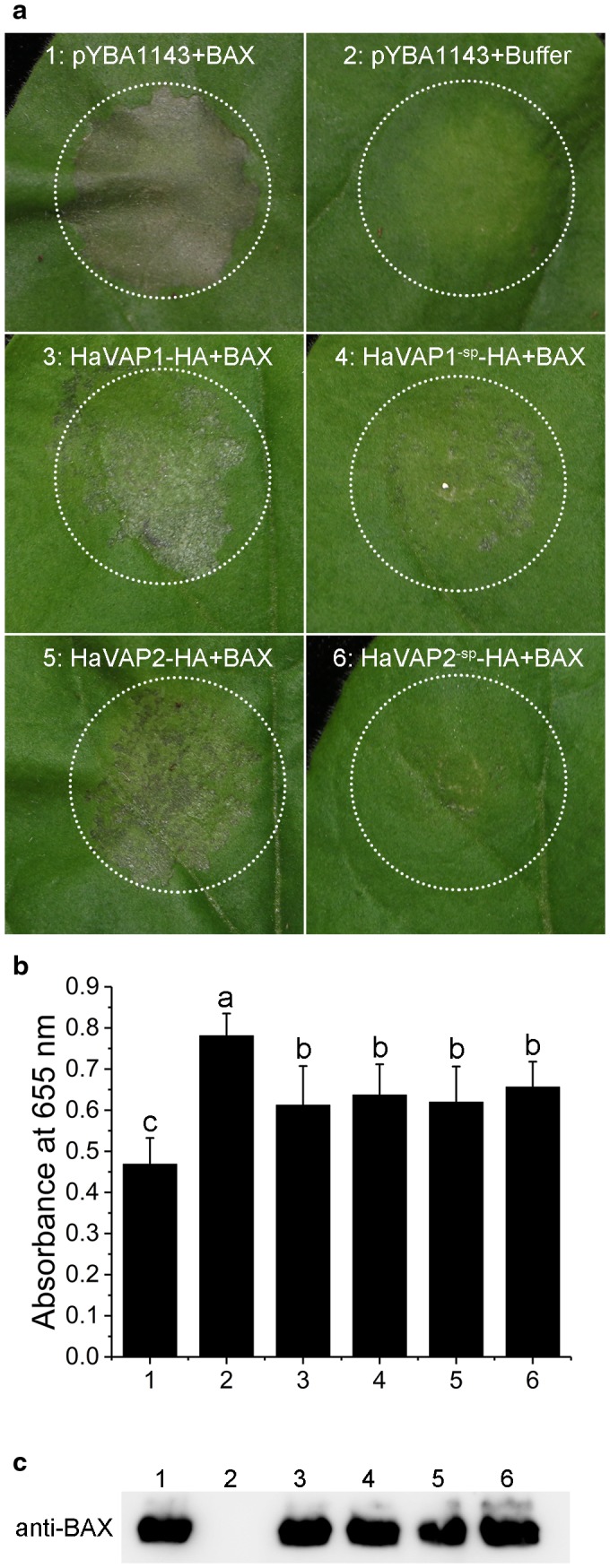
HaVAP1 and HaVAP2 suppress cell death induced by BAX in *Nicotiana benthamiana* leaves. (a) Phenotypes of leaves with infiltration of empty vector, HaVAP1‐HA, HaVAP1^‐sp^‐HA, HaVAP2‐HA or HaVAP2^‐sp^‐HA, followed by infiltration with BAX or infiltration buffer after 24 h. The photographs were captured at 3 days after infiltration. (b) Quantification of cell death by measurement of the absorbance of chlorophyll from infiltrated spots at a wavelength of 655 nm. The average numbers and bars represent the means and standard deviation, respectively. Different letters indicate significant differences according to Student’s *t*‐test, *P* < 0.05. (c) The expression of BAX was confirmed by western blotting. The numbers in (b) and (c) are the same combinations as in (a).

Cultures of *Agrobacterium* carrying *HaVAP1*, *HaVAP1^‐sp^*, *HaVAP2* and *HaVAP2^‐sp^* constructs, or the empty vector, were infiltrated into *N. benthamiana* leaves, and were followed 24 h later by the infiltration of *Agrobacterium* harbouring BAX or infiltration buffer. The empty vector resulted in significant necrosis at 3 days after the infiltration of BAX (Fig. 7a). In contrast, all four treatments with the constructs mentioned above showed milder necroses, especially the treatments without signal peptides (Fig. 7a). Infiltration with empty vector and infiltration buffer did not induce necrosis (Fig. 7a). The absorbance of chlorophyll at 655 nm was used to quantify cell death (Fig. 7b). Necroses resulted in low absorbance values. The expression of BAX was verified by western blotting (Fig. 7c). All of these results illustrate that both HaVAP1 and HaVAP2, with or without signal peptides, can suppress cell death induced by BAX.

### HaVAP2 physically interacts with a CYPRO4‐like protein from *H. vulgare*

**Figure 8:**
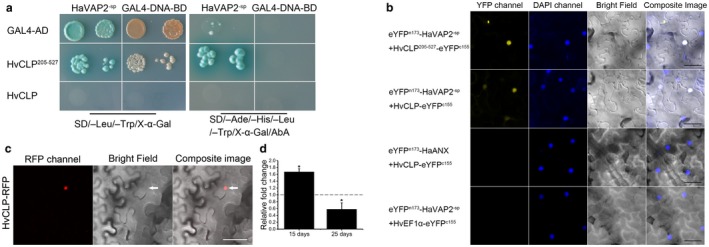
HaVAP2^‐sp^ interacts with HvCLP. (a) Interaction between HaVAP2^‐sp^ and HvCLP^205–527^ promotes the growth of yeast cells on selective quadruple dropout medium supplemented with X‐α‐Gal and Aureobasidin A (SD/‐Ade/‐His/‐Leu/‐Trp/X‐α‐Gal/AbA). The toxicity of full‐length HvCLP to yeast impedes the yeast two‐hybrid (Y2H) assay. Yeast cells containing HaVAP2^‐sp^ with Gal4 activation domain (GAL4‐AD) and yeast cells containing HvCLP^205–527^ with Gal4 DNA‐binding domain (GAL4‐DNA‐BD) fail to grow on SD/‐Ade/‐His/‐Leu/‐Trp/X‐α‐Gal/AbA medium. (b) The co‐expression of HaVAP2^‐sp^+HvCLP^205–527^ (top panel) and HaVAP2^‐sp^+HvCLP (second panel) reconstitutes enhanced yellow fluorescent protein (eYFP) in the nuclei of *Nicotiana benthamiana* leaves counterstained with the nucleic acid stain 4′,6‐diamidino‐2‐phenylindole (DAPI). The co‐expression of HaANX+HvCLP (third panel) and HaVAP2^‐sp^+HvEF1α (bottom panel) fails to reconstitute eYFP. The signals were collected at 2 days after agroinfiltration. Bars, 50 μm. (c) HvCLP was fused to the N‐terminus of a red fluorescent protein (RFP) reporter and accumulated in the nucleus (indicated with arrows) of an *N. benthamiana* leaf. The signal was collected at 3 days after agroinfiltration. Bars, 50 μm. (d) The transcript abundance of *HvCLP* decreased from 15 to 25 days after inoculation with *H. avenae*. Quantitative reverse transcription‐polymerase chain reaction (qRT‐PCR) assay was conducted to analyse the changes in transcript abundance in the infected plants relative to the abundance in non‐infected plants. The fold change values and bars represent the means and standard deviation, respectively. Asterisks (*) indicate significant differences according to Student’s *t*‐test, *P* < 0.01.

The different subcellular localizations between HaVAP1^‐sp^ and HaVAP2^‐sp^ mentioned above suggest different protein interaction patterns between HaVAP1 and HaVAP2 during *H. avenae* parasitism. To determine the differences between proteins that interact with HaVAP1 or HaVAP2 in the host, Y2H screening was conducted using the CDSs of HaVAP1 and HaVAP2 as bait without the signal peptides. Based on the prey library prepared from *H. vulgare* roots infected with *H. avenae*, six positive clones were identified with the bait of HaVAP1^‐sp^ (Table S1, see Supporting Information) or HaVAP2^‐sp^ (Table S2, see Supporting Information). As a result of the incomplete sequences of positive clones, we amplified the full‐length CDSs of all the positive clones for the verification of their interaction with HaVAP1^‐sp^ or HaVAP2^‐sp^ in yeast. None of six positive clones was verified to interact with HaVAP1^‐sp^. With regard to HaVAP2^‐sp^, one gene, which encodes a CYPRO4‐like protein (HvCLP for short, GenBank accession BAK04090.1), is toxic to yeast cells. However, a fragment containing a C‐terminal region of HvCLP (HvCLP^205–527^) was confirmed to specifically interact with HaVAP2^‐sp^ (Fig. 8a). The other five full‐length prey proteins showed no interaction with HaVAP2^‐sp^.

Subsequently, bimolecular fluorescence complementation (BiFC) assays were used to further test the interaction between HaVAP2^‐sp^ and HvCLP *in planta*. In these experiments, co‐expression of eYFP^n173^‐HaVAP2^‐sp^ and HvCLP^205–527^‐eYFP^c155^ in *N. benthamiana* leaves at 2 days after agroinfiltration reconstituted enhanced yellow fluorescent protein (eYFP) activity in the nuclei of infiltrated cells (first panel of Fig. 8b). The same fluorescence was observed after co‐expression of eYFP^n173^‐HaVAP2^‐sp^ and HvCLP‐eYFP^c155 ^(Fig. 8b, second panel). The co‐expression of eYFP^n173^‐HaANX/HvCLP‐eYFP^c155^ and eYFP^n173^‐HaVAP2^‐sp^/HvEF1α‐eYFP^c155^ did not reconstitute eYFP fluorescence (third and bottom panels of Fig. 8b). These BiFC assays further confirm the specific interaction between HaVAP2^‐sp^ and HvCLP *in planta*.

The interaction between two proteins within a plant cell necessitates the same cellular location of the two proteins. The subcellular localization and BiFC assays mentioned above suggest that HvCLP should be expressed in the nucleus, as is HaVAP2^‐sp^. To confirm this hypothesis, we conducted the fusion of HvCLP‐RFP to analyse the subcellular localization of HvCLP in *N. benthamiana*. As shown in Fig. 8c, HvCLP accumulated exclusively in the nucleus at 3 days after agroinfiltration. This result demonstrates that the cellular localization of HvCLP overlaps with the nuclear localization of HaVAP2^‐sp^, and suggests that these two proteins show physical association.

The developmental expression pattern indicates that *HaVAP2* expression increases sharply from par‐J2 to par‐J4 (Fig. 2d). Therefore, we infer that *HvCLP* expression will change in response to the increase in *HaVAP2* expression at the late stages. qRT‐PCR assay revealed that *HvCLP* expression decreased significantly from 15 to 25 days after inoculation (Fig. 8d). These data uncover an opposite pattern of developmental expression of *HvCLP* compared with *HaVAP2* during the late stages of parasitism.

## Discussion

The hosts of *H. avenae* are restricted to wheat, barley and certain other cereal crops. As a result of the difference in hosts, we speculate that different parasitic mechanisms are employed by *H. avenae* compared with *G. rostochiensis *and *H. schachtii*. In spite of the common biochemical properties of VAPs, the highly variable N‐ and C‐termini may result in different functions of these proteins (Wilbers *et al.*, 2018). In this study, we isolated and cloned the full‐length cDNA and gDNA of two *VAP* genes, *HaVAP1* and *HaVAP2*, from *H. avenae* based on an EST library (Cui *et al.*, 2018). To explore the functions of these two genes during *H. avenae* parasitism, experiments were carried out and the results are discussed below.

Phytonematode effectors are secreted by secretory organs, including subventral glands, dorsal glands, amphids and hypodermis/cuticle (Haegeman *et al.*, 2012). Among these, the subventral and dorsal glands, which connect with the stylet, are the most important components for secretion. *In situ* hybridization assays showed that *HaVAP1* was localized to the subventral oesophageal gland (Fig. 2a) and *HaVAP2* was localized to the dorsal oesophageal gland (Fig. 2b). The gene expression at each parasitic stage of phytonematodes is related to the role of the gene during parasitism. CWDEs, such as endoglucanases (Gao *et al.*, 2004; Hu *et al.*, 2013; Long *et al.*, 2013) and expansins (Liu *et al.*, 2016; Long *et al.*, 2012; Qin *et al.*, 2004), are expressed at the early stages of infection. Basal immunity inhibitors or inducers, such as MgGPP (Chen J *et al.*, 2017) and MiMAP‐1.2 (Castagnone‐Sereno *et al.*, 2009; Semblat *et al.*, 2001), also play an important role in the invasion of nematodes. The genes involved in host cell differentiation or auxin signal interference, such as *CLE* or *10A07*, accumulate at the late stages of infection (Chen S *et al.*, 2015; Guo *et al.*, 2011; Hewezi *et al.*, 2015; Lu *et al.*, 2009). qRT‐PCR assays showed that *HaVAP1* (Fig. 2c) was mainly expressed at the early stages, whereas *HaVAP2* (Fig. 2d) accumulated more strongly at the late stages. The differences in both tissue localization and developmental expression pattern suggest that different mechanisms may be employed by HaVAP1 and HaVAP2 during the parasitism of *H. avenae*.

Effectors should be secreted by nematodes before delivery into the host. Both HaVAP1 and HaVAP2 promoted RFP signals to accumulate in the extracellular space of *N. benthamiana* (Fig. 3), which verifies the secretion of these two proteins under the control of signal peptides. Lozano‐Torres *et al. *(2014) reported that both HsVAP1 and HsVAP2 increase the expression of *NPQ4*, which is localized in the chloroplasts of Arabidopsis and encodes a photosystem II subunit S protein involved in the regulation of singlet oxygen, which is used for redox‐dependent immune responses. The results in this study showed that HaVAP1^‐sp^ is expressed in chloroplasts (Fig. 4a), suggesting that HaVAP1 may be involved in the regulation of reactive singlet oxygen. Hs10A07 localizes to the nuclei of onion epidermal cells to modulate the auxin signal for syncytium development (Hewezi *et al.*, 2015). HaVAP2^‐sp^ is speculated to play a role in transcription based on its expression in the nucleus (Fig. 4b). No chloroplast transit peptide was detected in HaVAP1^‐sp^, suggesting that a non‐canonical transport mechanism is utilized by HaVAP1^‐sp^. A similar characteristic was found in HaVAP2^‐sp^, which lacks a nuclear localization sequence (NLS).

In most cases, effectors play a role in the promotion of the parasitism of phytonematodes (Davis *et al.*, 2008; Rosso *et al.*, 2011). However, some effectors are recognized as Avr proteins, which induce ETI directly or indirectly (Ali *et al.*, 2017). Silencing of *Cg‐1*, a candidate effector gene isolated from *M. javanica*, leads to virulence on tomato which carries the corresponding resistance gene *Mi‐1* (Gleason *et al.*, 2008). A chorismate mutase (Hg‐CM‐1) in *H. glycines* shows a similar behaviour to *Cg‐1*, although the corresponding resistance gene has not been identified (Bekal *et al.*, 2003; Lambert *et al.*, 2005). Lozano‐Torres *et al. *(2012) found that GrVAP1 acts as an Avr protein, identical to Avr2 in *Cladosporium fulvum*, which induces the resistance of tomato mediated by Cf‐2. The down‐regulation of *HaVAP1* facilitated the invasion of juveniles at early stages (Fig. 5). The developmental expression pattern indicated that *HaVAP1* is mainly expressed at pre‐J2 and par‐J2 (1 d) stages (Fig. 2c). All of these results suggest that HaVAP1 may enhance the resistance of the host at the early stages of invasion.

The persistence of RNAi in juveniles soaked in dsRNA always seems to be transitory, which makes the effect of RNAi on the genes expressed in the late stages limited (Rehman *et al.*, 2016). BSMV VIGS, a type of host‐induced gene silencing (HIGS), was used to confirm the function of HaVAP2 because of its constant generation of dsRNA (Yuan *et al.*, 2011). With regard to the efficiency of BSMV VIGS, a susceptible *T. aestivum *cultivar WEN19 was used as the host. The down‐regulation of *HaVAP2* inhibited the parasitism of *H. avenae* (Fig. 6). A previous study has confirmed that GrVAP1, HsVAP1 and HsVAP2 all promote the invasion of juveniles during the onset of parasitism by the modulation of basal immunity (Lozano‐Torres *et al.*, 2014). However, the difference in the developmental expression patterns of *HaVAP2* and the reported *VAPs* imply that a novel mechanism is used by HaVAP2.

BAX, a Bcl‐2 family protein, induces cell death in *N. benthamiana* leaves after its expression (Lacomme and Cruz, 1999). The suppression of cell death triggered by BAX is a typical method used to confirm the suppression of basal immunity by effectors (Wang *et al.*, 2011). Many effectors, such as Ha‐annexin (Chen C *et al.*, 2015), MiMsp40 (Niu *et al.*, 2016) and MeTCTP (Zhuo *et al.*, 2017), have been verified to suppress cell death triggered by BAX. Lozano‐Torres *et al. *(2014) reported that MiVAP1 and HsVAP1 inhibited cell death induced by INF1. In this study, both HaVAP1 and HaVAP2, with or without signal peptides, suppressed BAX‐induced cell death (Fig. 7). These two proteins may both suppress the basal immunity of the host.

The physical interactions between effectors and host receptors play important roles during parasitism (Chen S *et al.*, 2015; Guo *et al.*, 2011; Hewezi *et al.*, 2010; Huang *et al.*, 2006; Patel *et al.*, 2010; Rehman *et al.*, 2009). Y2H assay is a primary tool to screen target proteins from hosts using effectors as bait (Mitchum *et al.*, 2013). Based on the published genome of barley (*H. vulgare* L.) (The International Barley Genome Sequencing Consortium, 2012), the *H. vulgare* cultivar Golden Promise (Luo *et al.*, 2017) was used as a prey library. Although GrVAP1 interacts with Rcr3^pim^ and C14^tub^, two papain‐like cysteine proteases, to promote infections in tomato and potato (Lozano‐Torres *et al.*, 2012, 2014), the homologous protein HaVAP1^‐sp^ did not interact with any papain‐like cysteine protease from *H. vulgare* in Y2H assays. Although HaVAP2^‐sp^ activates the *MEL1* reporter gene and turns yeast colonies blue on selective double dropout medium supplemented with X‐α‐Gal (SD/‐Leu/‐Trp/X‐α‐Gal), *HIS3* and *ADE2* reporter genes remain inactive. Therefore, HaVAP2^‐sp^ can be used as a bait (Finley, 2007). A CYPRO4‐like protein (HvCLP) was isolated and a corresponding C‐terminal fragment was confirmed to interact with HaVAP2^‐sp^ (Fig. 8a).

The interaction between HaVAP2^‐sp^ and full‐length HvCLP was confirmed by BiFC and subcellular localization analyses. Similar to HaVAP2^‐sp^, HvCLP was also expressed in the nucleus (Fig. 8c), where their physical interaction occurred (Fig. 8b). It is reasonable that HaVAP2^‐sp^ is transferred into the nucleus to interact with HvCLP after secretion.

AtSBT3.14, a subtilase‐like serine protease, is suppressed by HsVAP1 and HsVAP2, contributing to infection (Lozano‐Torres *et al.*, 2014). qRT‐PCR assay indicated that the expression of *HvCLP* was inhibited at the late stage of parasitism (Fig. 8d). In addition to the observation that HaVAP2^‐sp^ interacted with HvCLP in the nucleus (Fig. 8b), and HaVAP2 accumulated strongly at the late stage of parasitism (Fig. 2d), it is suggested that the transcript abundance of *HvCLP* was probably suppressed by HaVAP2.

On the basis of the results described above, we infer that HaVAP1 suppresses basal innate immunity when transiently expressed in *N. benthamiana* and triggers immunity (ETI) during the parasitism of *H. avenae*. HaVAP2 is transferred into the nucleus to manipulate the defence response to promote the parasitism of *H. avenae*. Further study needs to be conducted to explain the role of HvCLP in the compatible interaction between *H. avenae* and *H. vulgare*. The identification of HaVAP1 and HaVAP2 broadens our understanding of VAPs that harbour SCP‐like domains, but employ different mechanisms during the parasitism of phytonematodes.

## Experimental Procedures

### Gene amplifications and sequence analyses

*Heterodera avenae* genomic DNA was isolated from pre‐J2s as described previously (Ou *et al.*, 2008). Total RNA of *H. avenae* was isolated from pre‐J2s using TRIzol reagent (Invitrogen, New York, NY, USA). DNase treatment and removal reagents (Ambion, Austin, TX, USA) were used to remove possible contaminating DNA from total RNA. Full‐length 5′ and 3′ ends of cDNA were obtained using a GeneRacer Kit (Invitrogen) combined with specific RACE primers. The gDNA and cDNA harbouring CDSs were amplified using PrimeSTAR HS DNA Polymerase (Takara, Shiga, Japan) with CDS primers.

The CDSs and conserved domains were analysed using ORF Finder (https://www.ncbi.nlm.nih.gov/orffinder/) and CD‐search (https://www.ncbi.nlm.nih.gov/Structure/cdd/wrpsb.cgi) from NCBI, respectively. Signal peptides were predicted using the SignalP 4.1 server (http://www.cbs.dtu.dk/services/SignalP/). The theoretical isoelectric point and molecular mass were computed using the Compute pI/Mw tool (http://web.expasy.org/compute_pi/) on ExPASy. The chloroplast transit peptide and NLS were predicted using the ChloroP 1.1 server (http://www.cbs.dtu.dk/services/ChloroP/) and the PSORT WWW server (https://psort.hgc.jp/), respectively. Homologous sequences were sought in the non‐redundant protein database using the BLASTp suite (https://blast.ncbi.nlm.nih.gov/Blast.cgi?PROGRAM=blastp&PAGE_TYPE=BlastSearch&LINK_LOC=blasthome) on NCBI. Sequence analyses and multiple sequence alignments were conducted using MEGA5.2 with ClustalW. The phylogenetic tree was computed based on full proteins using FastTree (version 2.1.10 No SSE3) with the Jones–Taylor–Thorton model.

### *In situ* hybridization

*In situ* hybridization was carried out using pre‐J2s, par‐J2s, par‐J3s and par‐J4s as described previously by de Boer *et al. *(1998). Single‐stranded probes were synthesized using a PCR DIG Probe Synthesis Kit (Roche, Indianapolis, IN, USA). Hybridization signals were detected using alkaline phosphatase‐conjugated anti‐DIG antibody and nitroblue tetrazolium chloride / 5‐bromo‐4‐chloro‐3‐indolyl‐phosphate (NBT/BCIP) substrate (DIG High Prime DNA Labeling and Detection Starter Kit II, Roche). Photographs were viewed and captured under an Olympus BX53 upright microscope (Olympus, Tokyo, Japan).

### Gene transcript abundance analyses

Eggs were isolated from the cysts of *H. avenae*. The pre‐J2s were hatched at 16 °C in the dark. The par‐J2s, par‐J3s and par‐J4s were isolated from roots infected by *H. avenae*. Adult males and females were separated from the soil around infected roots at 40 days after inoculation. The mRNA at each stage was isolated using a Dynabeads mRNA DIRECT Kit (Ambion). The SuperScript III First‐Strand Synthesis System for RT‐PCR (Invitrogen) was used to synthesize cDNA. Real‐time PCR was conducted using PowerUp SYBR Green Master Mix (Applied Biosystems, Foster City, CA, USA). *HaGAPDH* was used as a reference (Chen C *et al.*, 2015). The transcript abundances at the egg stage were used as the standard. Three independent replicates were conducted.

*Hordeum vulgare* roots at 15 and 25 days after inoculation with *H. avenae* were collected and ground to extract mRNA. *Hordeum vulgare* roots without inoculation at the same time points were used as control. The mRNA isolation, cDNA synthesis and real‐time PCR were carried out as mentioned above. *HvEF1α* (GenBank accession KP293845.1) was used as the reference (McGrann *et al.*, 2008). Three independent replicates were conducted.

### Subcellular localization analyses

*HaVAP1*, *HaVAP1^‐sp^*, *HaVAP2*, *HaVAP2^‐sp^* and *HvCLP* were constructed in pYBA1137 with TagRFP fused at the C‐terminus. All constructs, including empty vector pYBA1137, were transformed into *A. tumefaciens* EHA105 and cultured overnight. After precipitation, *Agrobacterium* cells were suspended in infiltration buffer containing 100 μm acetosyringone (Sigma‐Aldrich, St. Louis, MO, USA), 10 mm 2‐(*N*‐morpholino)ethanesulfonic acid (Sigma‐Aldrich) and 10 mm MgCl_2_ to reach an optical density at 600 nm (OD_600_) of 1.5. The *Agrobacterium* suspensions were kept at room temperature for 3–5 h and were then infiltrated into *N. benthamiana* leaves with a 1‐mL syringe. Three days after infiltration, the infiltrated leaves were collected and observed under a Zeiss LSM 880 laser confocal microscope (Zeiss, Jena, Germany). Plasmolysis and DAPI markers were performed by infiltrating a 30% glycerine–water solution and 1 μg/mL DAPI in *N. benthamiana* leaves, respectively. RFP, chlorophyll autofluorescence and DAPI signals were excited at 561, 488 and 405 nm, respectively, and collected at 586–647, 680–700 and 410–556 nm, respectively.

### *In vitro* RNAi

Four cDNA fragments of *HaVAP1* were amplified and purified using *H. avenae* cDNA as the template. An HiScribe T7 Quick High Yield RNA Synthesis Kit (NEB, Ipswich, MA, USA) was used to synthesize four dsRNAs based on the purified cDNA fragments of *HaVAP1*. The transcription reactions were purified using a MEGAclear Kit (Ambion). Then, 8000 pre‐J2s were soaked in dsRNA solution including 2 mg/mL dsRNA (purified above), 3 mm spermidine, 50 mm octopamine and 0.05% gelatin for 36 h to knock down *HaVAP1* in *H. avenae*. After soaking, approximately 500 treated pre‐J2s were inoculated onto each *H. vulgare* seedling. Five plants were inoculated for each treatment. Diethyl pyrocarbonate (DEPC)‐treated water was used as the control. Eight days after inoculation, the plant roots were stained with acid fuchsin, and the number of nematodes per plant was counted under a Leica M165c stereoscopic microscope (Leica, Wetzlar, Germany). The rest of the treated pre‐J2s were used to determine the efficiency of RNAi. The mRNA isolation, cDNA synthesis and real‐time PCR were carried out with the same methods as described in the ‘Gene transcript abundance analyses’ section. Four replicates were collected for each treatment.

### BSMV VIGS

The *HaVAP2* gene and *TaPDS* gene (GenBank accession FJ517553) fragments were constructed in pCa‐γbLIC to generate pCa‐γb:*HaVAP2* and pCa‐γb:*TaPDS* according to Yuan *et al. *(2011). The integrated constructs and empty vectors of pCaBS‐α and pCaBS‐β were transformed into *Agrobacterium* EHA105. After culture, *Agrobacterium *cells were precipitated and suspended in infiltration buffer (mentioned in the ‘Subcellular localization analyses’ section) to reach OD_600_ = 0.7. Then, *Agrobacterium* suspensions containing pCaBS‐α, pCaBS‐β and pCa‐γb:*HaVAP2* or pCa‐γb:*TaPDS* were mixed in equal amounts. The *Agrobacterium* suspensions were kept at room temperature for 3–5 h and then infiltrated into *N. benthamiana* leaves with a 1‐mL syringe. Twelve days after agroinfiltration, the infiltrated leaves were collected and ground in sodium phosphate buffer. The sap derived from *N. benthamiana* leaves was used to inoculate the leaves of *T. aestivum*. Ten plants were inoculated for each treatment. One thousand *H. avenae* pre‐J2s were inoculated onto each treated *T.* *aestivum* plant. Forty days after inoculation, the white adult females produced on the surfaces of the roots were collected to determine the efficiency of VIGS. The mRNA isolation, cDNA synthesis and real‐time PCR were carried out as described in the ‘Gene transcript abundance analyses’ section. pCa‐γb:*TaPDS* was used as the control. Four replicates were collected for each treatment. Ninety days after inoculation, the numbers of cysts and corresponding eggs per plant were counted under a Leica M165c stereoscopic microscope.

### Cell death analyses

*HaVAP1*, *HaVAP1^‐sp^*, *HaVAP2* and *HaVAP2^‐sp^* were constructed in pYBA1143 with a haemagglutinin (HA) tag fused at the C‐terminus. All constructs, including empty vector pYBA1143, were each transformed into *Agrobacterium *EHA105. After culture, *Agrobacterium *cells were precipitated and suspended in infiltration buffer (mentioned in the ‘Subcellular localization analyses’ section) to reach OD_600_ = 1.0. The *Agrobacterium* suspensions were kept at room temperature for 3–5 h, and then infiltrated into *N. benthamiana* leaves with a 1‐mL syringe. After 24 h, the *Agrobacterium *cells harbouring BAX or infiltration buffer were injected into the same infiltration site. The infiltrated leaves were collected and photographed at 3 days after the infiltration of BAX. A 13‐mm‐diameter disc was cut from each infiltrated spot and immersed in 0.5 mL of dimethylformamide (Sigma‐Aldrich) overnight. After incubation, 150 μL of extract was transferred to a 96‐well plate for absorbance measurement at 655 nm. Six replicates were conducted. Western blotting was employed to confirm the expression of BAX. Total proteins of infiltrated leaves were extracted using a Plant Protein Extraction Kit (CWBio, Beijing, China). Protein crude extracts were separated by sodium dodecylsulfate‐polyacrylamide gel electrophoresis (SDS‐PAGE) and transferred to a polyvinylidene difluoride (PVDF) membrane. After blocking, the membrane was incubated with an anti‐BAX antibody (Sigma‐Aldrich) to bind BAX. Horseradish peroxidase (HRP)‐conjugated goat anti‐mouse immunoglobulin G (IgG) (Sigma) was used as the secondary antibody. Proteins were visualized using an Immobilon Western Chemiluminescent HRP Substrate (Millipore, Billerica, MA, USA).

### Y2H assays

The cDNA of *H. vulgare *cultivar Golden Promise roots infected by *H. avenae* was used to construct a prey library in *Saccharomyces* *cerevisiae* strain Y187. *HaVAP1^‐sp^* and *HaVAP2^‐sp^* were cloned into pGBKT7 to generate pGBKT7:*HaVAP1^‐sp^* and pGBKT7:*HaVAP2^‐sp^*. These two integrated constructs were transformed into *S. cerevisiae* strain Y2HGold to generate bait strains. After testing the bait for autoactivation and toxicity, two‐hybrid library screenings were performed according to the user manual of the Matchmaker Gold Yeast Two‐Hybrid System (Clontech, Mountain View, CA, USA). The prey plasmids were extracted and sequenced to search for the corresponding intact CDSs on NCBI (https://www.ncbi.nlm.nih.gov/) or International Barley Sequencing Consortium (http://www.public.iastate.edu/~imagefpc/IBSC%20Webpage/IBSC%20Template-home.html) databases. The CDSs were cloned into pGADT7 and then transformed into Y187 for positive interaction confirmation.

### BiFC assays

*HaVAP2^‐sp^* and *HaANX* (*Ha‐annexin*, GenBank accession KJ562871.1) were constructed in pSPYNE(R)173. *HvCLP^205–527^*, *HvCLP* and *HvEF1α* (GenBank accession KP293845.1) were constructed in pSPYCE(M) (Waadt *et al.*, 2008). All constructs were transformed into *Agrobacterium *EHA105. After culture, *Agrobacterium *cells were precipitated and suspended in infiltration buffer (mentioned in the ‘Subcellular localization analyses’ section) to reach OD_600_ = 1.0. Then, the *Agrobacterium* suspensions were mixed as pairs in equal amounts: HaVAP2^‐sp^/HvCLP^205–527^, HaVAP2^‐sp^/HvCLP, HaANX/HvCLP and HaVAP2^‐sp^/HvEF1α. The *Agrobacterium* suspensions were kept at room temperature for 3–5 h and then infiltrated into *N. benthamiana* leaves with a 1‐mL syringe. Two days after infiltration, the infiltrated leaves were collected and observed under a Zeiss LSM 880 laser confocal microscope. DAPI markers were introduced by infiltrating 1 μg/mL DAPI in *N. benthamiana* leaves. The eYFP and DAPI signals were excited at 514 and 405 nm, respectively, and collected at 519–620 and 410–508 nm, respectively.

### Data analyses and primers

The qRT‐PCR data were analysed by the 2^–ΔΔCt^ method (Livak and Schmittgen, 2001). The average values and standard deviations were calculated using Microsoft Excel 2016. Statistical significances were determined using Student’s *t*‐test. All charts were generated by Origin 2017.

All the primers used in this study are listed in Table S3 (see Supporting Information).

## Accession Numbers

*HaVAP1* GenBank accession MH255798, *HaVAP2* GenBank accession MH255799.

## Supporting information


**Fig. S1  **Sequence analyses of HaVAP1. (a) The full‐length cDNA of *HaVAP1*. The untranslated regions (UTRs) are in bold, the start and stop codons are underlined and the four introns are presented in lower‐case letters. (b) The amino acid sequence of HaVAP1. The underlined letters at the N‐terminus indicate a predicted signal peptide; the putative SCP‐like extracellular protein domain is in bold.

**Fig. S2  **Sequence analyses of HaVAP2. (a) The full‐length cDNA of *HaVAP2*. The untranslated regions (UTRs) are in bold, the start and stop codons are underlined and the four introns are presented in lower‐case letters. (b) The amino acid sequence of HaVAP2. The underlined letters at the N‐terminus indicate a predicted signal peptide; the putative SCP‐like extracellular protein domain is in bold.

**Table S1  **Candidate proteins that interact with HaVAP1.

**Table S2  **Candidate proteins that interact with HaVAP2.

**Table S3  **Primers used in this study.
