# Supplementary material for: Two venom allergen‐like proteins, HaVAP1 and HaVAP2, are involved in the parasitism of Heterodera avenae
Source: Mol Plant Pathol. 2019 Jan 9;20(4):471–84. doi: 10.1111/mpp.12768 (PMC6637866; doi:10.1111/mpp.12768)
Supplement: Supplementary file 1 — Fig. S1 Sequence analyses of HaVAP1. (a) The full‐length cDNA of HaVAP1. The untranslated regions (UTRs) are in bold, the start and stop codons are underlined and the four introns are presented in lower‐case letters. (b) The amino acid sequence of HaVAP1. The underlined letters at the N‐terminus indicate a predicted signal peptide; the putative SCP‐like extracellular protein domain is in bold. [file MPP-20-471-s001.pdf]

a

AAAGGAACAATGAGACCATTCATTAAAGTCTTGTTATAAAAATTCATCACTTATTTCT
AATTTTTAATATTAAAATTGGCTAAACCCATTCGAAAATGGAACTCCACTCAAAAGTT
GTCTGCTTCTTCTTGCTCATTGCCCTAATTGgtatttaagaaaatttaatttttaaatattaactttttccccgg
agggttcatggtgaaaatgcaaaacagaaaatttctcatttctcagCTATTCCTTACAATGTTTGGGCACTTT
CTGCTGGCGGTCGCGTGTCGGTGCTTAATTGTCACAACAATTATCGTTCCCAATTGG
CCAAAGGCACTGCGGACAACAAATCCGGCAAAATGCCGGCTGGTAGCAATTTGATAG
AGCTAAAATATTTGAATGAACATGAGAAGGGTGCACAGAGCTGGGCCGACGGTTGCT
CAATGTCCCACTCGAGTTCATCACAACGCCAAGGGATGGGCGAGAATTTGTACATGT
CAAGTTCATCTACCATTTCGGAAGgttagcacgacggacaaatgctttgttgtataattaagcaaataaagca
taataacacagaaaaaacattgggatttttcttgggacaacagtagccatttctttaattacttttgaataaacccccgcaataa
tttttgtttaatccattttgaactctgattttagaatttattttttgaaaaaaacagggtttactatttaagCTGAGGCGCTCAA
ACAAGCTTGTGACATGTGGTGGGCAGAGCTCAAAGAGTTTGGCTTTGACCAAAGCC
TGGTGCTGAACATGAACGAGTTCAACAAGGGCATCGGACATTGGAGCCAGgcaaaattg
cttattttttggtcaattaatgaataatttgtttgacttttgcagCAAGCGTGGGCCAAAACTGCTCAAATTGGT
TGTGCCTTGGCACGATGTCCCAGCTCCCAATGGCAAACATGGGTTGTGTGCCGCTA
CAAAGCGGCgtaagtggatgaattttagcacatttatgagacatttattaatatataagtaagaaatcgggaaatattgtt
gaatatttataatttttaacttcatttttatgcctttttatcatattcagGGGAAATATGTTGAACGAAATGGTCTAC
AAAAAGGGAACTGCATGCAGTGGCTGTTCAGATTATTCTGGTGCATCGTGCAATAATG
CCAATGGTCTGTGCGTTGTGCCATGA**GTGACGCGCTGAGCAGTTTTCGAATTGGGG**
AATAGATGAAAAAAGAGTTGGAGAAATTTTTATAAAGGAAATTTACCAAATTAAAGT
GCATTAAATGAATTGTTAATCATTTAATTAAATTGAAAAAATAAATTTCATTTGTTAAA
AAAAAAAAAAAAAAAAAAAAA

b

MELHSKVVCFFLLIALIAIPYNVWALSAGGRVS**VLNCHNNYRSQLAKGTADNKSGKMPA**
GSNLIELKYLNEHEKGAQSWADGCSMSHSSSSQRQGMGENLYMSSSSTISEAEALKQ
ACDMWWAELKEFGFDQSLVLNMNEFNKGIGHWSQQAWAKTAQIGCALARCPSSQW
QTWVVCRYKAAGNMLNEMVYKKGTACSGCSDYSGASCNNANGLCVVP
