# Supplementary material for: Two venom allergen‐like proteins, HaVAP1 and HaVAP2, are involved in the parasitism of Heterodera avenae
Source: Mol Plant Pathol. 2019 Jan 9;20(4):471–84. doi: 10.1111/mpp.12768 (PMC6637866; doi:10.1111/mpp.12768)
Supplement: Supplementary file 2 — Fig. S2 Sequence analyses of HaVAP2. (a) The full‐length cDNA of HaVAP2. The untranslated regions (UTRs) are in bold, the start and stop codons are underlined and the four introns are presented in lower‐case letters. (b) The amino acid sequence of HaVAP2. The underlined letters at the N‐terminus indicate a predicted signal peptide; the putative SCP‐like extracellular protein domain is in bold. [file MPP-20-471-s002.pdf]

a

GGTTTAATTACCCAAGTTTGAGATATATTAAAATGGGTCATCGTGGTTTAATGTTCATT
TTTGGGTTTATTTTTGgtaagaattgcaaatgttattttgtaaatatttatttccctacaaaatttcctttccaagGTTTT
TTTTTGCTCAATTCGGCGGCACTGCTCAGCCAAGACGAGCAGAACTATTGTGCCACC
TACAACAACGATCTTCGCCGAAATGTGGCCACAGGCAATCAGCCGAACAGAAATGGC
ATGTTGCCGCCCGCCACGAACATGTTGCAGATGTTCTACAACACGACGACCGGCCA
AACTGCCCAGgcaagacactttttggaccagctagtacagtacccttattttagccgatttatatatttcgataatttttgga
ccagaattgagttccggaaaatttttgcaatcgaaattccggcaattttaatgtccagtgaaaacataaaattgaatcgcgtag
aactgaatgaatcatgcacattcccacagcagACATGGGCCAATCAATGCAAATTTGTGCACTCAAA
TGAACCCGGCGGGTTGGGCGAAAACATTTACTACTCCACGGCGGAAGGGATGAGCA
ACAgtaaacttgggatttccataagaaaatgtaaatttcgaaaaatagCTGAAGCATTGAACAAAGCCTTC
GGCTATTGGTGGAATGAGTCAATTACCGTTGGCATTCCCGAAAATTTGGTGGTGGAC
CAAAGCAACTTCGGGCCAATTGGGCATTTCACGCAGATGGCATGGCACAGTACGAC
AGACATTGGGTGTGGCGTGGCCAGCTGCCAAAATGAAGAATGGAAAACCTTTGTTGT
CTGCAATTATTGGCCACCgtaataattcatattaaacaaattaatttaaattccagtcgtcagcgggtgtctataggc
gccgcctgggacaaattgtttgtttttgaaacgcaaatgattcattataaaaagcaaaaacactaagattttatttggaaaaaat
tatttttatagatttgccgatgaaaaatttttatttaattaaaaagatacaattggcaaaacaatggtttaaaaaaatacatattttct
cagaaatttggtcggaacttctgtctgcgccttgtttctttcggtcggaatcctccaatttgttttgccttaaaaagtattcggcgccc
tttaaacctactcaccccaaacgcctaagacactttaagaattcaaaaagtaattccagtaaaacagaattaattgtggtgcc
aaatgaacgaatgtcttcttgtcagcataccatttgatatttttctaccaattcggttcagAGGAAATTACGTTGGCGA
AACGGTGTATGGCGCGGGTGCCACGTGCAGTGCATGCGACCCGGGATGGTCATGT
GACAAGGGGAGGAGGGGCCTTTGTGTGCGCAACGGCAAAAAGTGA**CAGCTAAGGC**
ACAAAAAAGGAGGAGAAAAGCGGCACAAAAAAGGAGGGGAAAGGAAAAGCGGG
CGGAAAAGGACAAACAAGTGATACCTTTGAAATGACGAAAAACCATTTGGATGATA
ATTTTATAAATTATATAGTGGAGAAGTGAAGCAGCAACGACAAAAATGTAAAACAAT
TTCAAAAAAAAAAAAAAAAA

b

MGHRGLMFIFGFIFGFFLLNSAALLSQDEQNYCATYN**NDLRRNVATGNQPNRNGMLPP**
ATNMLQMFYNTTTGQTAQTWANQCKFVHSNEPGGLGENIYYSTAEGMSNTEALNKAF
GYWWNESITVGIPENLVVDQSNFGPIGHFTQMAWHSTTDIGCGVASCQNEEWKTFVVC
NYWPPGNYVGETVYGAGATCSACDPGWSCDKGRRGLCVRNGKK
