# Supplementary material for: Two venom allergen‐like proteins, HaVAP1 and HaVAP2, are involved in the parasitism of Heterodera avenae
Source: Mol Plant Pathol. 2019 Jan 9;20(4):471–84. doi: 10.1111/mpp.12768 (PMC6637866; doi:10.1111/mpp.12768)
Supplement: Supplementary file 3 — Table S1 Candidate proteins that interact with HaVAP1. [file MPP-20-471-s003.docx]

Table S1 Candidate proteins that interact with HaVAP1

| No. | Accession | Description | Database | Clone length / Gene length |
| --- | --- | --- | --- | --- |
| 1 | CAD66657.1 | putative cysteine proteinase precursor | NCBI | 202-377 / 1-377 |
| 2 | HORVU1Hr1G051200.12 | hyperosmolality-gated ca2+ permeable channel | IBSC | 545-689 / 1-689 |
| 3 | BAK07803.1 | kelch repeat-containing f-box family expressed | NCBI | 405-523 / 1-523 |
| 4 | BAJ90137.1 | cytosolic malate dehydrogenase | NCBI | 152-333 / 1-333 |
| 5 | HORVU0Hr1G009080.5 | copper-transporting ATPase PAA2, chloroplastic | IBSC | 580-761 / 1-761 |
| 6 | AGI97132.1 | R2R3-type Myb transcription factor | NCBI | 1-249 / 1-392 |

NCBI: National Center for Biotechnology Information

IBSC: International Barley Sequencing Consortium

The sequence lengths of every clone and corresponding gene were indicated in the last column
