# Supplementary material for: Two venom allergen‐like proteins, HaVAP1 and HaVAP2, are involved in the parasitism of Heterodera avenae
Source: Mol Plant Pathol. 2019 Jan 9;20(4):471–84. doi: 10.1111/mpp.12768 (PMC6637866; doi:10.1111/mpp.12768)
Supplement: Supplementary file 4 — Table S2 Candidate proteins that interact with HaVAP2. [file MPP-20-471-s004.docx]

Table S2 Candidate proteins that interact with HaVAP2

| No. | Accession | Description | Database | Clone length / Gene length |
| --- | --- | --- | --- | --- |
| 1 | BAJ87072.1 | dihydroxy-acid dehydratase-like | NCBI | 150-441 / 1-595 |
| 2 | HORVU1Hr1G051200.12 | hyperosmolality-gated Ca2+ permeable channel | IBSC | 545-689 / 1-689 |
| 3 | HORVU3Hr1G038580.1 | nedd8-activating enzyme e1 catalytic subunit-like | IBSC | 241-455 / 1-455 |
| 4 | BAK04090.1 | CYPRO4-like protein | NCBI | 234-527 / 1-527 |
| 5 | HORVU2Hr1G026340.3 | pyrrolidone-carboxylate peptidase family expressed | IBSC | 105-219 / 1-219 |
| 6 | HORVU7Hr1G080800.2 | ubiquitin-conjugating enzyme e2 | IBSC | 35-148 / 1-148 |

NCBI: National Center for Biotechnology Information

IBSC: International Barley Sequencing Consortium

The sequence lengths of every clone and corresponding gene were indicated in the last column
