# Supplementary material for: Two venom allergen‐like proteins, HaVAP1 and HaVAP2, are involved in the parasitism of Heterodera avenae
Source: Mol Plant Pathol. 2019 Jan 9;20(4):471–84. doi: 10.1111/mpp.12768 (PMC6637866; doi:10.1111/mpp.12768)
Supplement: Supplementary file 5 — Table S3 Primers used in this study. [file MPP-20-471-s005.docx]

Table S3 Primers used in this study

| Primer | Sequence (5' to 3' end) | References |
| --- | --- | --- |
| HaVAP1race5 | GATTTGTTGTCCGCAGTGCCTTTG | This study |
| HaVAP1race3 | GCCAAAGGCACTGCGGACAACAAA |  |
| HaVAP2race5 | CTTGGCTGAGCAGTGCCGCCGAAT |  |
| HaVAP2race3 | AATTACGTTGGCGAAACGGTGTATG |  |
| HaVAP1cdsf | ATGGAACTCCACTCAAAAGTTG |  |
| HaVAP1cdsr | TCATGGCACAACGCACAGAC |  |
| HaVAP2cdsf | ATGGGTCATCGTGGTTTAATGT |  |
| HaVAP2cdsr | TCACTTTTTGCCGTTGCGCAC |  |
| HaVAP1hybf | TCGAGTTCATCACAACGCCA |  |
| HaVAP1hybr | CTTTGTAGCGGCACACAACC |  |
| HaVAP2hybf | CTCAGCCAAGACGAGCAGAA |  |
| HaVAP2hybr | AAATGTTTTCGCCCAACCCG |  |
| HaVAP1qf | CTACAAAGCGGCGGGAAAT |  |
| HaVAP1qr | ATGCACCAGAATAATCTGAACAGC |  |
| HaVAP2qf | CACTGCTCAGCCAAGACG |  |
| HaVAP2qr | CCACATTTCGGCGAAGATC |  |
| GAPDHqf | AGCGGCACAGAACATCATCC | Chen *et al*., 2015 |
| GAPDHqr | GGTCCTCCGTGTAGCCCAAA |  |
| HaVAP1Bam | TAGAACTAGTGGATCATGGAACTCCACTCAAAAGTTGTC | This study |
| HaVAP1^-sp^Bam | TAGAACTAGTGGATCATGCTTTCTGCTGGCGGTCGCGTGTCG |  |
| HaVAP1Hind | CGGTATCGATAAGCTTTGGCACAACGCACAGACCATTGGC |  |
| HaVAP2Bam | TAGAACTAGTGGATCATGGGTCATCGTGGTTTAATGTTC |  |
| HaVAP2^-sp^Bam | TAGAACTAGTGGATCATGCTGCTCAGCCAAGACGAGCAGAAC |  |
| HaVAP2Hind | CGGTATCGATAAGCTTCTTTTTGCCGTTGCGCACACAAAG |  |
| HvCLPBam | TAGAACTAGTGGATCATGGGCGCCGCCAACAGCCGCGAG |  |
| HvCLPHind | CGGTATCGATAAGCTTACGTCTGTTTGCGATGCTGAAGGA |  |
| HaVAP1^-sp^EcoR | GGAGGCCAGTGAATTCCTTTCTGCTGGCGGTCGCGTGTCG |  |
| HaVAP1BamH | CGAGCTCGATGGATCCTGGCACAACGCACAGACCATTGGC |  |
| HaVAP2^-sp^EcoR | GGAGGCCAGTGAATTCCTGCTCAGCCAAGACGAGCAGAAC |  |
| HaVAP2BamH | CGAGCTCGATGGATCCCTTTTTGCCGTTGCGCACACAAAG |  |
| HvCLPEcoR | GGAGGCCAGTGAATTCATGGGCGCCGCCAACAGCCGC |  |
| HvCLP^205^EcoR | GGAGGCCAGTGAATTCATGCGCGGCGAGACCAATATGCTG |  |
| HvCLPBamH | CGAGCTCGATGGATCCACGTCTGTTTGCGATGCTGAAGGA |  |
| HaVAP2^-sp^Bam | GCCTACTAGTGGATCCCTGCTCAGCCAAGACGAGCAGAAC |  |
| HaVAP2Xho | GAGCGGTACCCTCGAGCTTTTTGCCGTTGCGCACACAAAG |  |
| HvCLPXba | CACGGGGGACTCTAGAATGGGCGCCGCCAACAGCCGC |  |
| HvCLP^205^Xba | CACGGGGGACTCTAGAATGCGCGGCGAGACCAATATGCTG |  |
| HvCLPCla | TCGACAGTACTATCGATACGTCTGTTTGCGATGCTGAAGGA |  |
| HaANXBam | GCCTACTAGTGGATCCATGATGTCTAACGCAACCAAAAAC |  |
| HaANXXho | GAGCGGTACCCTCGAGGTTCCCTTTGATCAGTGTCAACA |  |
| HvEF1αXba | CACGGGGGACTCTAGAATGGGTAAGGAGAAGACTCACATC |  |
| HvEF1αCla | TCGACAGTACTATCGATTTTCTTCTTGATGGCAGCCTTGGT |  |
| dsRNA1f | TAATACGACTCACTATAGGGGCTCATTGCCCTAATTGCTATTC |  |
| dsRNA1r | TAATACGACTCACTATAGGGTACAAATTCTCGCCCATCCCTT |  |
| dsRNA2f | TAATACGACTCACTATAGGGGCTTTGACCAAAGCCTGGTGC |  |
| dsRNA2r | TAATACGACTCACTATAGGGATTTCGTTCAACATATTTCCCGC |  |
| dsRNA3f | TAATACGACTCACTATAGGGGCCCTAATTGCTATTCCTTACAA |  |
| dsRNA3r | TAATACGACTCACTATAGGGATTCTCGCCCATCCCTTGGCG |  |
| dsRNA4f | TAATACGACTCACTATAGGGGCCTGGTGCTGAACATGAACG |  |
| dsRNA4r | TAATACGACTCACTATAGGGTAATCTGAACAGCCACTGCATG |  |
| HaVAP2VIGSf | AAGGAAGTTTAACGGCTATTGGTGGAATGAGT |  |
| HaVAP2VIGSr | AACCACCACCACCGTGCCAACGTAATTTCCTGGTG |  |
| TaPDSVIGSf | AAGGAAGTTTAATTTCTCCAGGAGAAG | Yuan *et al*., 2011 |
| TaPDSVIGSr | AACCACCACCACCGTTCTCCAGTTATTTGAG |  |
| HvCLPqf | TGGGCACCACAGACACATAC | This study |
| HvCLPqr | CGCCCACTGAATCCAGTCTT |  |
| HvEF1αqf | ATGATTCCCACCAAGCCCAT | McGrann *et al*., 2008 |
| HvEF1αqr | ACACCAACAGCCACAGTTTGC |  |
